# Modulation of EGFR Activity by Molecularly Imprinted Polymer Nanoparticles Targeting Intracellular Epitopes

**DOI:** 10.1021/acs.nanolett.3c01374

**Published:** 2023-10-30

**Authors:** Stanislav S. Piletsky, Ekaterina Baidyuk, Elena V. Piletska, Larissa Lezina, Konstantin Shevchenko, Donald J. L. Jones, Thong H. Cao, Rajinder Singh, Alan C. Spivey, Eric O. Aboagye, Sergey A. Piletsky, Nickolai A. Barlev

**Affiliations:** † Department of Chemistry, Imperial College London, Molecular Sciences Research Hub, White City Campus, London W12 0BZ, United Kingdom; ‡ L.A. Orbeli Institute of Physiology NAS, Yerevan 0028, Republic of Armenia; § School of Chemistry, University of Leicester, Leicester LE1 7RH, United Kingdom; ∥ Department of Cancer Studies, University of Leicester, Leicester LE1 7RH, United Kingdom; ⊥ Institute of Cytology, 197101 Saint-Petersburg, Russia; # Leicester Cancer Research Centre, University of Leicester, Leicester Royal Infirmary, Leicester LE1 7RH, United Kingdom; ∇ Department of Cardiovascular Sciences, University of Leicester, Leicester LE1 7RH, United Kingdom; ○ National Institute for Health Research, Leicester Biomedical Research Centre, Glenfield Hospital, Leicester LE1 7RH, United Kingdom; ⧫ Department of Surgery and Cancer, Imperial College London, Hammersmith Campus, Du Cane Road, London SW7 2BX, United Kingdom; ¶ Nazarbayev University School of Medicine, 53 Kabanbay Batyr Ave, Nur-Sultan 010000, Republic of Kazakhstan; + Sechenov First Medical University, 119992 Moscow, Russia

**Keywords:** cancer, molecularly imprinted polymers, epidermal growth factor receptor, nanoparticles, epitopes

## Abstract

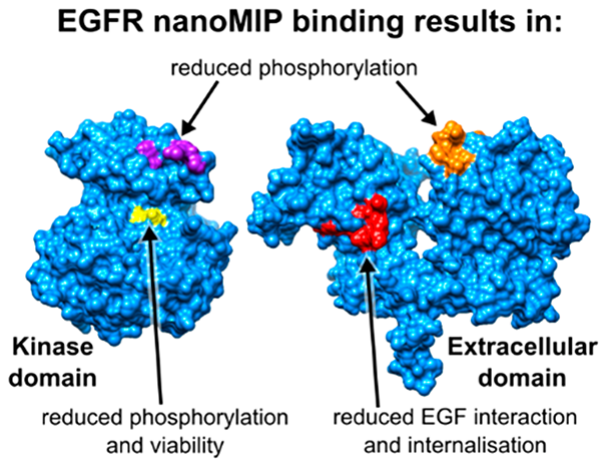

In recent years, molecularly imprinted polymer nanoparticles (nanoMIPs) have proven to be an attractive alternative to antibodies in diagnostic and therapeutic applications. However, several key questions remain: how suitable are intracellular epitopes as targets for nanoMIP binding? And to what extent can protein function be modulated via targeting specific epitopes? To investigate this, three extracellular and three intracellular epitopes of epidermal growth factor receptor (EGFR) were used as templates for the synthesis of nanoMIPs which were then used to treat cancer cells with different expression levels of EGFR. It was observed that nanoMIPs imprinted with epitopes from the intracellular kinase domain and the extracellular ligand binding domain of EGFR caused cells to form large foci of EGFR sequestered away from the cell surface, caused a reduction in autophosphorylation, and demonstrated effects on cell viability. Collectively, this suggests that intracellular domain-targeting nanoMIPs can be a potential new tool for cancer therapy.

One of the main challenges involved in the development of new cancer therapies is the identification and validation of tractable targets. Plasma membrane proteins are attractive targets, due to both their accessibility and the key roles they play in the abnormal signal transduction processes required for carcinogenesis.^1^ One notable and clinically relevant example of a plasma membrane protein with roles in the diagnosis and progression of cancer is the epidermal growth factor receptor (EGFR). EGFR is a 180 kDa transmembrane protein that is subdivided into three sub domains: a highly glycosylated extracellular domain (comprising amino acids 1–621), a single transmembrane domain (amino acids 622–644), and a cytoplasmic domain (amino acids 645–1186) which has intrinsic tyrosine kinase activity. Activation of EGFR results from the binding of growth factors, such as epidermal growth factor (EGF), transforming growth factor alpha (TGF-α), and amphiregulin, which induce receptor homo- and/or heterodimerization and stimulation of the intrinsic receptor tyrosine kinase activity.^2^ This promotes autophosphorylation of tyrosine residues within the cytoplasmic domain of the receptor, providing docking sites for a variety of adaptor proteins and enzymes involved in the recruitment and activation of downstream intracellular-signaling cascades, including the mitogen-activated protein kinase (MAPK) and phosphatidylinositol-3-kinase (PI-3K) pathways.^3^ These signaling cascades can promote proliferation, angiogenesis, and invasion and inhibit apoptosis, key mechanisms underlying tumor growth and progression.^4^ This oncogenic potential in conjunction with the aberrant expression and/or activation of EGFR, which has been reported in a wide range of human malignancies, provides a strong rationale for targeting this growth factor receptor.^5,6^

Currently there are two distinct groups of therapeutic agents employed for targeting EGFR in cancer treatment. These are monoclonal antibodies (mAbs) that bind to extracellular domains and small molecule tyrosine kinase inhibitors, such as gefitinib and erlonitinib, that target the intracellular TK domain.^7^ The response rate in clinical studies for these agents varies from 5% to 24%.^7^ Commercial, highly successful anti-EGFR monoclonal antibodies such as cetuximab (Erbitux, C225) and panitumumab (Vectibix) bind the extracellular ligand-binding domain III of the receptor, blocking ligand-binding receptor activation, phosphorylation, and downstream receptor signaling, and, to some extent, induce receptor internalization and degradation. Tyrosine kinase inhibitors (TKIs) are typically adenosine triphosphate (ATP) analogues, capable of inhibiting EGFR signaling by occupying ATP binding pockets on the intracellular catalytic kinase domain of the receptor. As in the case of monoclonal antibodies, they act by preventing autophosphorylation and activation of several downstream signaling pathways.^8^ In contrast to antibodies, TKIs are less specific and have higher toxicity and a shorter lifetime (<48 h).^7^ The use of monoclonal antibodies against EGFR targets is highly successful; however it is limited in general due to the following factors:^9−13^

- High production costs and poor stability
- Poor oral bioavailability
- Immunogenicity exhibited by all therapeutic mAbs currently in clinical practice
- Resistance of certain tumors to anti-EGFR mAb therapy
- EGFR dimerization induced by mAb binding, which can lower the threshold for ligand activation
- Significant morbidity caused by mAbs used in cancer chemotherapy
- Ethical issues associated with the use of animals in antibody production

Therefore, developing synthetic ligands capable of binding to different EGFR epitopes and preventing autophosphorylation and other downstream pathways would be of great value for the treatment of drug-resistant cancers. In a previous study, we prepared nanoMIPs with specificity for a linear peptide of EGRF (amino acids 418–425). This peptide is located within the extracellular domain of EGFR and overlaps with the extracellular EGF binding region.^14^ In these studies, nanoMIPs were used for the targeted delivery of doxorubicin to EGFR-overexpressing cells (MDA-MB-468), with the intention of triggering apoptosis. However, in contrast to mAbs, EGFR-nanoMIPs without doxorubicin had no effect on the survival of MDA-MB-468 cells, indicating that simply binding nanoMIPs to EGFR was not sufficient to affect the cell viability. Further investigation was then necessary to determine whether targeting specific epitopes would generate nanoMIPs with inherent antitumor properties and whether intracellular epitopes were suitable as targets. These nanoMIPs would be more akin to therapeutic antibodies and would not require their conjugation with a cytotoxic agent for generating antitumor effects.

It is a reasonable assumption that epitopes that are suitable for mAb production might not be appropriate for nanoMIP production, as proteins and polymers differ both in size and the type of functional groups used in molecular recognition. We have recently developed an experimental approach for using molecular imprinting to identify peptide sequences on the protein surface with potential “antigenic” properties.^15^ This method involved the synthesis of MIPs in the presence of whole protein, followed by partial proteolysis of the protein bound to polymer and subsequent sequencing of peptides bound to the polymer. We have previously shown the success of this approach with targets such as KRAS and acetylcholine esterase (AChE).^16,17^ This technique has been modified for the characterization of surface proteins of whole cells, an approach which was dubbed “snapshot imprinting”.^18^ In this previous work, snapshot imprinting was performed for two cell lines, HN5 and MDA-MB-468, generating a list of epitopes with the potential to serve as good targets for nanoMIP binding. In the current work, we synthesized nanoMIPs for six epitopes of EGFR identified during snapshot imprinting. Three of these epitopes were from the extracellular domain of EGFR, and three were intracellular, with two epitopes belonging to the kinase domain (responsible for phosphorylation) and one from the epidermal growth factor (EGF) binding domain. These nanoMIPs were characterized and tested for their ability to induce cell apoptosis in the absence of any supplementary chemotherapeutic agent.

During snapshot imprinting, 36 peptides belonging to EGFR were identified among the two cell lines. Among these were peptides from both the extracellular and intracellular domains. No epitopes were identified from the transmembrane domain (622–644), likely due to poor accessibility caused by the cell membrane. Taking into account that some peptides were subsections of larger peptides and that several peptides overlapped, 18 EGFR sequences were identified as possible epitopes, which were compared to EGFR epitopes listed on the Immune Epitope Database and Analysis Resource (IEDB; https://www.iedb.org/home_v3.php; Figure 1). Three epitopes belonging to the extracellular domain of EGFR were previously synthesized and tested for their ability to bind to their target epitope peptide and to whole EGFR, but this work stopped short of assessing intracellular targets or cellular response.^18,19^ Herein, we expand this work to include the imprinting of both extracellular and intracellular epitopes and characterization of downstream effects following cellular binding. Seven EGFR peptides were selected for the preparation of the MIPs and further testing, listed below (Table 1). Each peptide featured a terminal cysteine for immobilization and glycine to act as a spacer, listed below in parentheses.

**Figure 1:**
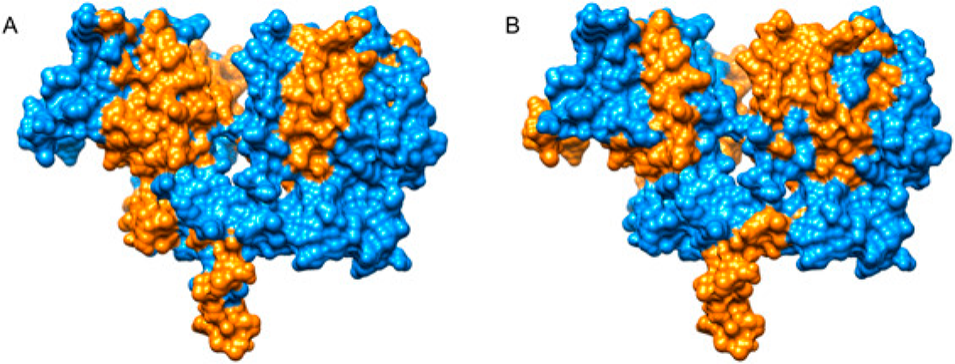
Extracellular region of EGFR. Regions considered to be epitopes highlighted in orange and the remainder in blue. Epitopes selected from (A) IEDB and (B) snapshot imprinting.^18^

**Table 1:** Epitopes of EGFR Used for the Generation of nanoMIPs

nanoMIP	template	sublocation
MIP-0	(CG)TKGKLQSGF	N/A (scrambled sequence)
MIP-1	(CG)KLFGTSGQK	extracellular
MIP-2	(CG)GMNYLEDR	intracellular (kinase domain center)
MIP-3	(CG)GVLGSGAFGTVYK	intracellular (kinase domain edge)
MIP-4	(CG)NLQEILHGAVR	extracellular
MIP-5	(CG)MHLPSPTDSNFYR	intracellular
MIP-6	(CG)LTQLGTFEDHFLSLQR	extracellular (EGF binding domain)

As described above, the autophosphorylation of EGFR following ligand binding leads to a number of downstream effects critical for cell proliferation. The effects of EGFR-imprinted nanoMIPs on autophosphorylation were investigated via the treatment of MDA-MB-468 cells followed by Western blot analysis. Five autophosphorylation sites have been identified in EGFR, all of which are clustered at the extreme carboxyl-terminus encompassing the final 194 amino acids.^20^ Among these phosphorylation sites, tyrosine (Tyr)1068 and Tyr1173 were investigated in this work. In particular, we focused on the possible effect of nanoMIPs on the phosphorylation state of Tyr-1068 (Y1068), the most proximal phosphorylated Tyr residue of the kinase domain, and Tyr-1173 (Y1173), the most distal phosphorylated residue. Certain nanoMIPs, specifically MIP-0, MIP-1, and MIP-4, caused an increase in autophosphorylation in the absence of exogenous EGF (Figure 2A). Given the high level of phosphorylation observed following treatment with the non-EGFR specific MIP-0, it appears likely that phosphorylation was not caused by specific nanoMIP binding. Nonspecific interactions with nanoparticles have been previously shown to induce autophosphorylation of EGFR.^21,22^ The lowest levels of pY1068 phosphorylation (2-fold reduction compared to the nontreated control) were observed for MIP-2 and MIP-3 (Figure 2A, upper panel). As described in Table 1, MIP-2 and MIP-3 were imprinted for peptides that make up part of the kinase domain of EGFR that is responsible for autophosphorylation. In contrast with pY1068 results, the same samples stained with antibodies against phosphorylated pY1173 showed a much wider range of effects from strong attenuation for MIP-1, -3, and -4; medium or no attenuation for MIP-2, -5, and -6; and robust activation by nonspecific MIP-0 (Figure 2A, lower panel). This result indicates that phosphorylation of EGFR at Y1173 in the absence of a stimulatory ligand can be the consequence of nonspecific association of MIPs with the kinase domain.

**Figure 2:**
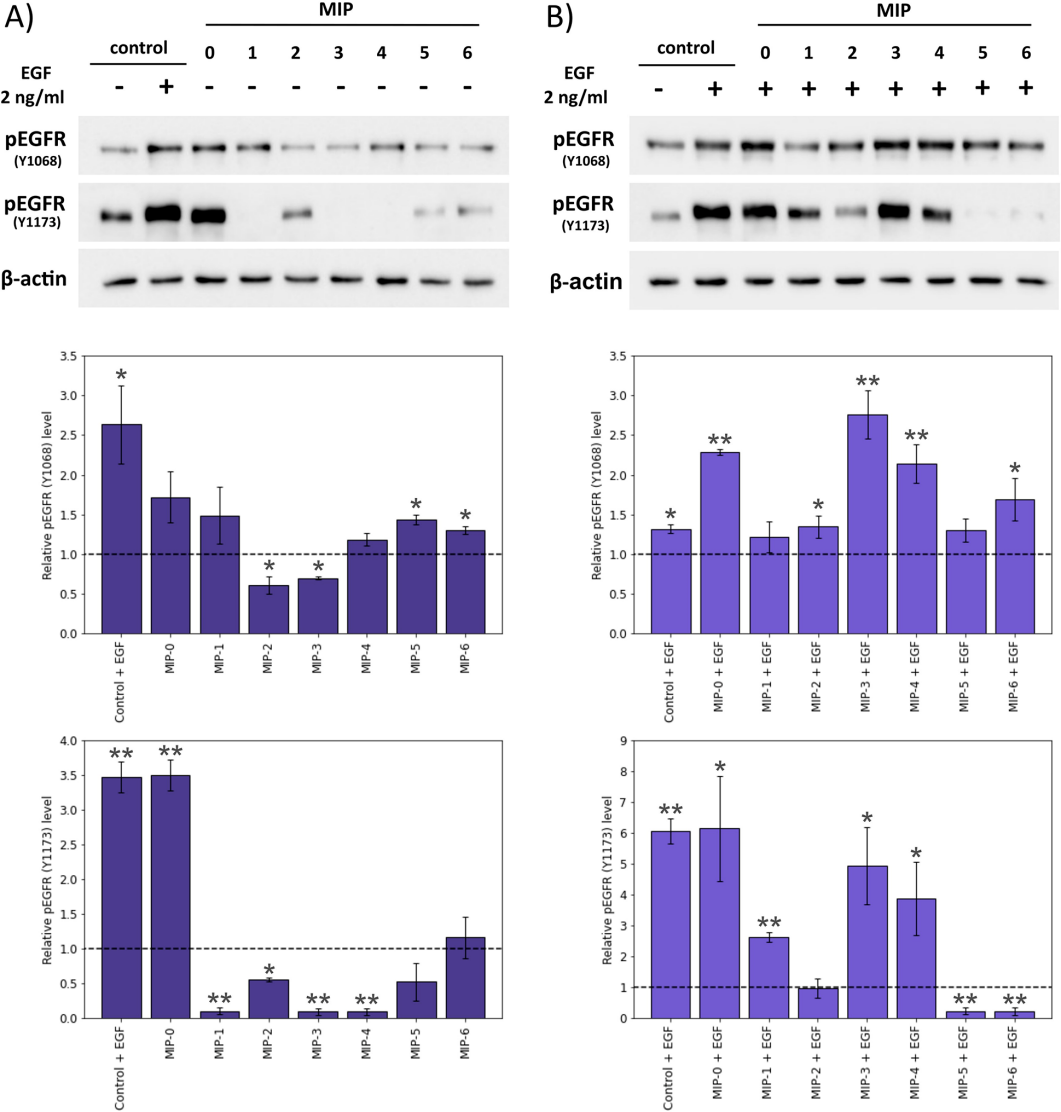
Western blot analysis demonstrating the effects of nanoMIP binding on phosphorylation of EGFR before (A) and after (B) EGF treatment. The average values for three independent experiments are presented (mean ± SEM), with comparisons performed using a randomization test.^23^ The statistical significance of differences between the control MIP nontreated sample and other MIP-treated samples is indicated, *P < 0.05, **P < 0.01.

In order to observe whether MIP binding can interfere with EGF-induced dimerization and phosphorylation, cells were treated with MIPs and then subsequently with EGF (Figure 2B). Cells treated with MIP-1 showed a slightly reduced level of phosphorylation in comparison to MIP-0, which did not prevent EGF-induced phosphorylation (Figure 2B, upper panel). The epitope used for imprinting MIP-1 was the most abundant epitope found during snapshot imprinting and is found on the extracellular domain of EGFR.^18^ It is speculated that MIP-1 competes with EGF to bind to EGFR, reducing the rate of phosphorylation. Two other MIPs directed against the extracellular domain of EGFR (MIP-4 and MIP-6) showed the opposite results: whereas MIP-4 did not prevent autophosphorylation on Tyr1068 and only slightly reduced the level of pY1173, the effect of MIP-6 on EGF-induced autophosphorylation of EGFR was much more pronounced, which is consistent with the fact that MIP-6 displayed high affinity for EGFR (a low dissociation constant (*K*_d_) for recombinant EGFR) during SPR analysis (Table S1). Furthermore, treatment of MDA-468 cells (high EGFR) with MIP-6 attenuated their proliferation rate 2-fold (Figure S3). At the same time, the proliferation of SKBR-3 cells (low EGFR) was almost unaffected (85% of the control level). The reason why MIP-4, another extracellular domain-directed nanoMIP, did not affect the Y1068 autophosphorylation is likely because it has low affinity for EGFR, i.e., high *K*_d_*in vitro* (Table S1). Variations in *K*_d_ between MIP-1, -4, and -6 may be due to differences in the conformation of isolated, recombinant EGFR compared to EGFR in a cellular environment. It has previously been observed that EGFR exists as an inactivated dimer even prior to ligand binding.^24^ The existence of alternative conformations may also explain why epitopes that are abundant during snapshot imprinting may not have strong interactions with the isolated protein during SPR analysis.

Remarkably, when the effect on autophosphorylation at Y1068 and Y1173 was assessed for MIPs directed against the intracellular domain of EGFR (MIP-2, -3, and -5), we found a strong correlation between their *K*_d_ values and the ability of MIPs to inhibit autophosphorylation. Specifically, high affinity MIP-2 and MIP-5 that displayed the lowest values of *K*_d_ (0.2 and 11 nM, respectively, Table S1) were able to robustly down-regulate autophosphorylation (∼6- and 12-fold reduction compared to MIP-0) as judged by the pY1173 signal. On the other hand, MIP-3, which has a relatively higher *K*_d_ (22 nM) failed to significantly affect EGFR autophosphorylation. As mentioned earlier, MIP-2 and MIP-3 were imprinted for peptides that make up part of the kinase domain of EGFR and MIP-5 was imprinted for a peptide that is sequentially adjacent to the phosphorylated Y1068 tyrosine residue. Although an exact explanation of this phenomenon has yet to be determined, we speculate that binding of MIPs to either the kinase domain or the residue of phosphorylation prevents phosphorylation as a result of steric interference of the relatively large nanoMIP.

To further investigate how nanoMIP binding affects the intracellular fate of EGFR in the absence of EGF stimulation, MDA-MB-468 cells treated with three types of EGFR-nanoMIP (MIP-1, MIP-2, and MIP-5) were subsequently stained with EGFR-specific antibodies and DAPI to highlight the nuclei. As expected, cells not treated with nanoMIPs or EGF showed primarily surface staining with anti-EGFR antibodies (Figure 3A). Cells treated with EGF showed significant staining of internalized EGFR (Figure 3B). This is to be expected, as EGF binding results in dimerization and internalization of EGFR. Interestingly, treatment with MIP-1 failed to cause internalization of EGFR. In contrast, treatment with MIP-2 and to a lesser extent MIP-5 results in a visible increase in the number and size of EGFR-containing foci in the cytoplasm (Figure 3E,F,G).

**Figure 3:**
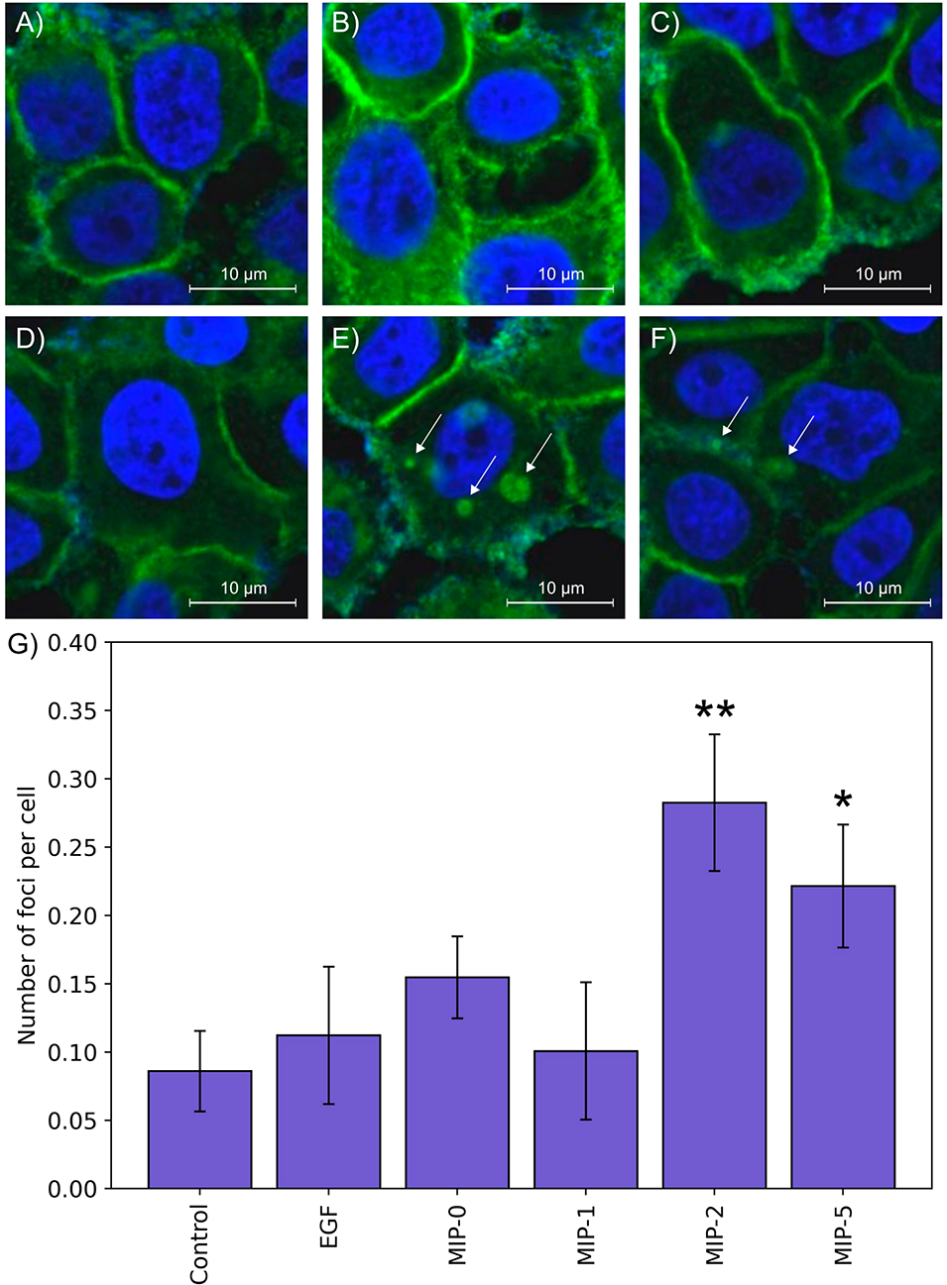
Confocal microscopy of MDA-MB-468 cells stained with anti-EGFR antibody: (A) nontreated control cells; (B) cells treated with EGF. (C–F) Cell were treated with different MIPs prior to EGF-treatment: (C) MIP-0, (D) MIP-1, (E) MIP-2, (F) MIP-5. Scale bars = 10 μm. (G) Morphometric analysis of the number of EGFR foci in MDA-MD-468 cells after treatment with various types of MIPs. Arrows indicate cytoplasmic EGFR foci. The foci of at least 100 cells were counted for each condition; error bars depict SEM of number of foci of different fields of view. The statistical significance of differences between the control MIP nontreated sample and other MIP-treated samples is indicated, *P < 0.05, **P < 0.01.

Given that these nanoMIPs were prepared using identical monomer mixtures and differed only in their template peptide, this difference in behavior is presumably due to differences in their binding profile. MIP-1 is imprinted with an extracellular sequence of EGFR, and MIP-2 and MIP-5 are imprinted with intracellular sequences. It therefore seems likely that binding of MIP-1 may result in competition with EGF, and so a lower degree of dimerization and subsequent internalization. It has been previously demonstrated that a key downstream effect of EGFR phosphorylation is ubiquitination of EGFR on multiple sites by ubiquitin ligase.^25^ This ubiquitination is necessary for the efficient degradation of EGFR following internalization.^26^ It therefore seems probable that blocking of the initial autophosphorylation of EGFR results in a reduction in the rate at which the kinase domain phosphorylates ubiquitin ligase, leading to an increase in internalized EGFR in foci. Alternatively, it is possible that other downstream pathways are disrupted by the presence of intracellularly bound nanoMIPs. For example, the epitope used for imprinting of MIP-2 is adjacent to tyrosine residue Y845, the phosphorylation of which plays a role in a variety of functions including cell proliferation, cell cycle control, mitochondrial regulation of cell metabolism, and gamete activation.^27^ Further work is necessary to identify the exact cause of this behavior, including additional phosphorylation studies at other residues and quantification of other protein concentrations.

Finally, MIPs imprinted against the intracellular domain of EGFR (MIP-2 and MIP-5) were assessed for their effect on cell survival. To this end, we used two cell lines that are known to drastically differ in their levels of EGFR expression: MDA-MB-468 (high levels of EGFR) and MCF-7 (low levels of EGFR).^28,29^ Cells were incubated with MIP-2 or MIP-5 for 12 h at various concentrations (Figure 4). Of these four samples, MIP-2 showed the most pronounced effect on cell viability, resulting in a ∼20% reduction in viability of MDA-MB-468 following incubation with 100 μg mL^–1^ of MIP-2. These nanoMIPs had a less pronounced effect on the viability of low-EGFR cell line MCF7 (Figure 4). This implies that MIP-2, potentially acting on the kinase domain of EGFR, can selectively inhibit the growth of cell lines only with high expression levels of EGFR (Figure S4). This occurs even in the absence of a chemotherapeutic agent, indicating that via careful selection of epitopes, nanoMIPs may be able to act as drugs in their own right, in the absence of a therapeutic payload. MIP-5 conversely resulted in a minor increase in viability, the reasons for this increase are uncertain. It is possible that binding to certain domains of the protein can affect its conformation, resulting in greater accessibility for phosphorylation and other post-translational modification, which can lead to enhanced proliferation.

**Figure 4:**
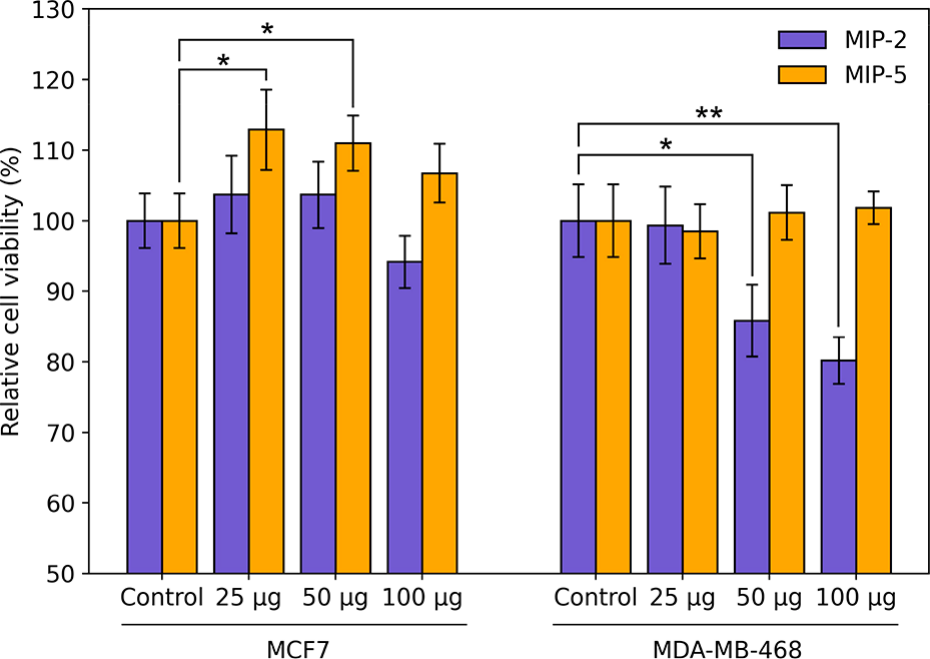
Effect of EGFR-specific MIPs on the cell viability of MDA-MB-468 and MCF7. Error bars show standard deviation across experiments performed in triplicate. * - *P* < 0.05, ** - *P* < 0.01.

Collectively, careful selection of epitopes for imprinting using the snapshot imprinting technique allowed us to generate nanoMIPs against the intracellular kinase domain of EGFR, and binding to these epitopes allowed us to modulate this signaling cascade and hence cancer cell survival. Despite having the same monomeric composition, nanoMIPs imprinted with different epitopes showed great differences in their induced effects following binding. This work serves to demonstrate the potential for nanoMIPs to target the intracellular domains of relevant biological molecules, thereby acting directly as therapeutic agents rather than only as delivery agents. In general, it should be possible to generate nanoMIPs with a diverse spectrum of effects that may affect protein–protein interactions (PPI), enzymatic activities (e.g., phosphorylation), intracellular localization of target proteins, and more. Future *in vivo* studies should define whether nanoMIPs can be used not only as chemical probes for specific cellular processes but also as therapeutics for precision medicine.

## Supporting Information Available

Additional experimental details, materials, and methods; cellular uptake study; physical characterization of nanoMIPs (PDF)

## References

[ref1] Pedersen M. W.; Jacobsen H. J.; Koefoed K.; Hey A.; Pyke C.; Haurum J. S.; Kragh M. Sym004: A Novel Synergistic Anti-Epidermal Growth Factor Receptor Antibody Mixture with Superior Anticancer Efficacy. Cancer Res. 2010, 70, 588 10.1158/0008-5472.CAN-09-1417. 20068188

[ref2] Burden S.; Yarden Y. Neuregulins and Their Receptors: A Versatile Signaling Module in Organogenesis and Oncogenesis. Neuron 1997, 18 (6), 847–855. 10.1016/S0896-6273(00)80324-4. 9208852

[ref3] Lemmon M. A.; Schlessinger J. Cell Signaling by Receptor-Tyrosine Kinases. Cell 2010, 141 (7), 1117 10.1016/j.cell.2010.06.011. 20602996 PMC2914105

[ref4] Salomon D. S.; Brandt R.; Ciardiello F.; Normanno N. Epidermal Growth Factor-Related Peptides and Their Receptors in Human Malignancies. Critical Reviews in Oncology and Hematology 1995, 19 (3), 183–232. 10.1016/1040-8428(94)00144-I. 7612182

[ref5] Nicholson R. I.; Gee J. M. W.; Harper M. E. EGFR and Cancer Prognosis. Eur. J. Cancer 2001, 37, 9 10.1016/S0959-8049(01)00231-3. 11597399

[ref6] Baselga J.; Rischin D.; Ranson M.; Calvert H.; Raymond E.; Kieback D. G.; Kaye S. B.; Gianni L.; Harris A.; Bjork T.; Averbuch S. D.; Feyereislova A.; Swaisland H.; Rojo F.; Albanell J. Phase I Safety, Pharmacokinetic, and Pharmacodynamic Trial of ZD1839, a Selective Oral Epidermal Growth Factor Receptor Tyrosine Kinase Inhibitor, in Patients with Five Selected Solid Tumor Types. J. Clin Oncol 2002, 20 (21), 4292–4302. 10.1200/JCO.2002.03.100. 12409327

[ref7] Seshacharyulu P.; Ponnusamy M. P.; Haridas D.; Jain M.; Ganti A. K.; Batra S. K. Targeting the EGFR Signaling Pathway in Cancer Therapy. Expert Opin Ther Targets 2012, 16 (1), 15–31. 10.1517/14728222.2011.648617. 22239438 PMC3291787

[ref8] Ciardiello F. Epidermal Growth Factor Receptor Tyrosine Kinase Inhibitors as Anticancer Agents. Drugs 2000, 60, 25–32. 10.2165/00003495-200060001-00003. 11129169

[ref9] Ecker D. M.; Jones S. D.; Levine H. L. The Therapeutic Monoclonal Antibody Market. MAbs 2015, 7 (1), 9–14. 10.4161/19420862.2015.989042. 25529996 PMC4622599

[ref10] Berg T. Modulation of Protein-Protein Interactions with Small Organic Molecules. Angew. Chem., Int. Ed. Engl. 2003, 42 (22), 2462–2481. 10.1002/anie.200200558. 12800163

[ref11] Cochran A. G. Protein-Protein Interfaces: Mimics and Inhibitors. Curr. Opin Chem. Biol. 2001, 5 (6), 654–659. 10.1016/S1367-5931(01)00262-9. 11738175

[ref12] Gadek T. R.; Nicholas J. B. Small Molecule Antagonists of Proteins. Biochem. Pharmacol. 2003, 65 (1), 1–8. 10.1016/S0006-2952(02)01479-X. 12473372

[ref13] Sharma S. K.; Ramsey T. M.; Bair K. W. Protein-Protein Interactions: Lessons Learned. Curr. Med. Chem. Anticancer Agents 2002, 2 (2), 311–330. 10.2174/1568011023354191. 12678748

[ref14] Canfarotta F.; Lezina L.; Guerreiro A.; Czulak J.; Petukhov A.; Daks A.; Smolinska-Kempisty K.; Poma A.; Piletsky S.; Barlev N. A. Specific Drug Delivery to Cancer Cells with Double-Imprinted Nanoparticles against Epidermal Growth Factor Receptor. Nano Lett. 2018, 18 (8), 4641–4646. 10.1021/acs.nanolett.7b03206. 29969563

[ref15] Piletsky S.; Piletska E.; Canfarotta F.; Jones D. Methods and Kits for Determining Binding Sites. GB1704823.2, October 4, 2018. https://patents.google.com/patent/WO2018178629A1/ewebn (accessed February 5, 2020).

[ref16] Norman R. L.; Singh R.; Muskett F. W.; Parrott E. L.; Rufini A.; Langridge J. I.; Runau F.; Dennison A.; Shaw J. A.; Piletska E.; Canfarotta F.; Ng L. L.; Piletsky S.; Jones D. J. L. Mass Spectrometric Detection of KRAS Protein Mutations Using Molecular Imprinting. Nanoscale 2021, 13 (48), 20401–20411. 10.1039/D1NR03180E. 34854867 PMC8675027

[ref17] Piletsky S. A.; Bedwell T. S.; Paoletti R.; Karim K.; Canfarotta F.; Norman R.; Jones D. J. L.; Turner N. W.; Piletska E. v. Modulation of Acetylcholinesterase Activity Using Molecularly Imprinted Polymer Nanoparticles. J. Mater. Chem. B 2022, 10 (35), 6732–6741. 10.1039/D2TB00278G. 35355036

[ref18] Piletsky S. S.; Piletska E.; Poblocka M.; Macip S.; Jones D. J. L.; Braga M.; Cao T. H.; Singh R.; Spivey A. C.; Aboagye E. O.; Piletsky S. A. Snapshot Imprinting: Rapid Identification of Cancer Cell Surface Proteins and Epitopes Using Molecularly Imprinted Polymers. Nano Today 2021, 41, 101304 10.1016/j.nantod.2021.101304.

[ref19] Piletsky S. S.; Garcia Cruz A.; Piletska E.; Piletsky S. A.; Aboagye E. O.; Spivey A. C. Iodo Silanes as Superior Substrates for the Solid Phase Synthesis of Molecularly Imprinted Polymer Nanoparticles. Polymers (Basel) 2022, 14 (8), 1595 10.3390/polym14081595. 35458345 PMC9026888

[ref20] Wang F.; Wang S.; Wang Z.; Duan J.; An T.; Zhao J.; Bai H.; Wang J. Phosphorylated EGFR Expression May Predict Outcome of EGFR-TKIs Therapy for the Advanced NSCLC Patients with Wild-Type EGFR. J. Exp Clin Cancer Res. 2012, 31 (1), 65 10.1186/1756-9966-31-65. 22901364 PMC3548765

[ref21] Peuschel H.; Sydlik U.; Grether-Beck S.; Felsner I.; Stöckmann D.; Jakob S.; Kroker M.; Haendeler J.; Gotić M.; Bieschke C.; Krutmann J.; Unfried K. Carbon Nanoparticles Induce Ceramide- and Lipid Raft-Dependent Signalling in Lung Epithelial Cells: A Target for a Preventive Strategy against Environmentally-Induced Lung Inflammation. Part Fibre Toxicol 2012, 9, 48 10.1186/1743-8977-9-48. 23228165 PMC3546038

[ref22] Stöckmann D.; Spannbrucker T.; Ale-Agha N.; Jakobs P.; Goy C.; Dyballa-Rukes N.; Hornstein T.; Kümper A.; Kraegeloh A.; Haendeler J.; Unfried K. Non-Canonical Activation of the Epidermal Growth Factor Receptor by Carbon Nanoparticles. Nanomaterials 2018, 8 (4), 267 10.3390/nano8040267. 29690640 PMC5923597

[ref23] Nuzzo R. L. Randomization Test: An Alternative Analysis for the Difference of Two Means. PM&R 2017, 9 (3), 306–310. 10.1016/j.pmrj.2017.02.001. 28237692

[ref24] Purba E. R.; Saita E. I.; Maruyama I. N. Activation of the EGF Receptor by Ligand Binding and Oncogenic Mutations: The “Rotation Model. Cells 2017, 6 (2), 13 10.3390/cells6020013. 28574446 PMC5492017

[ref25] Frey M. R.; Dise R. S.; Edelblum K. L.; Polk D. B. P38 Kinase Regulates Epidermal Growth Factor Receptor Downregulation and Cellular Migration. EMBO J. 2006, 25 (24), 5683–5692. 10.1038/sj.emboj.7601457. 17139251 PMC1698902

[ref26] Longva K. E.; Blystad F. D.; Stang E.; Larsen A. M.; Johannessen L. E.; Madshus I. H. Ubiquitination and Proteasomal Activity Is Required for Transport of the EGF Receptor to Inner Membranes of Multivesicular Bodies. J. Cell Biol. 2002, 156 (5), 843–854. 10.1083/jcb.200106056. 11864992 PMC2173306

[ref27] Sato K.-i. Cellular Functions Regulated by Phosphorylation of EGFR on Tyr845. Int. J. Mol. Sci. 2013, 14 (6), 10761–10790. 10.3390/ijms140610761. 23702846 PMC3709701

[ref28] Lev D. C.; Kim L. S.; Melnikova V.; Ruiz M.; Ananthaswamy H. N.; Price J. E. Dual Blockade of EGFR and ERK1/2 Phosphorylation Potentiates Growth Inhibition of Breast Cancer Cells. Br. J. Cancer 2004, 91 (4), 795 10.1038/sj.bjc.6602051. 15280923 PMC2364773

[ref29] Jeong H.; Kim J.; Lee Y.; Seo J. H.; Hong S. R.; Kim A. Neuregulin-1 Induces Cancer Stem Cell Characteristics in Breast Cancer Cell Lines. Oncol. Rep. 2014, 32 (3), 1218–1224. 10.3892/or.2014.3330. 25018110

