# Supplementary material for: Modulation of EGFR Activity by Molecularly Imprinted Polymer Nanoparticles Targeting Intracellular Epitopes
Source: Nano Lett. 2023 Oct 30;23(21):9677–82. doi: 10.1021/acs.nanolett.3c01374 (PMC10636853; doi:10.1021/acs.nanolett.3c01374)
Supplement: Supplementary file 1 — nl3c01374_si_001.pdf [file nl3c01374_si_001.pdf]

Modulation of EGFR Activity by Molecularly Imprinted Polymer Nanoparticles Targeting Intracellular Epitopes

Stanislav S. Piletsky,[1] Ekaterina Baidyuk,[2,3] Elena V. Piletska,[4] Larissa Lezina,[5] Konstantin Shevchenko,[2] Donald J. L. Jones,[6,7,8] Thong H. Cao,[7,8] Rajinder Singh,[6] Alan C. Spivey,[1] Eric O. Aboagye,[9] Sergey A. Piletsky[4] and Nickolai A. Barlev,*[10]*

1 Stanislav S. Piletsky, Alan C. Spivey
Department of Chemistry
Imperial College London
Molecular Sciences Research Hub, White City Campus, London, W12 0BZ, UK
E-mail: stanislav.piletsky14@imperial.ac.uk

2 Ekaterina Baidyuk, Konstantin Shevchenko
Institute of Cytology,
Saint-Petersburg Russia

3 Ekaterina Baidyuk
L.A. Orbeli Institute of Physiology NAS,
Yerevan, Republic of Armenia

4 Elena V. Piletska, Sergey A. Piletsky
School of Chemistry
University of Leicester
Leicester, UK

5 Larissa Lezina
Department of Cancer Studies
University of Leicester
Leicester, UK

6 Donald J. L. Jones, Rajinder Singh
Leicester Cancer Research Centre
University of Leicester
Leicester Royal Infirmary, Leicester, UK

7 Donald J. L. Jones, Thong H. Cao
Department of Cardiovascular Sciences
University of Leicester
Leicester, UK

8 Donald J. L. Jones, Thong H. Cao
National Institute for Health Research
Leicester Biomedical Research Centre
Glenfield Hospital Leicester UK

9 Eric O. Aboagye
Department of Surgery and Cancer
Imperial College London
Hammersmith Campus, Du Cane Road, London, UK

10 Nickolai A Barlev
Sechenov 1st Medical University, Moscow, Russia;
Nazarbayev University School of Medicine
53 Kabanbay Batyr Ave, Nur-Sultan 010000, Republic of Kazakhstan
E-mail: nikolai.barlev@nu.edu.kz

S1. Materials and Methods

Methods

Materials

All reagents and solvents were purchased from Merck Life Science UK Ltd (Gillingham, UK) and Fisher Scientific Ltd (Loughborough, UK) unless otherwise stated. Microglass beads (50-100 µm) were purchased from Microbeads AG, Switzerland. Peptides were synthesised by ZheJiang Ontores Biotechnologies Co. Ltd. (Hangzhou, China).

HN5 and MCF7 cells were provided by the Division of Radiotherapy and Imaging & Cancer Therapeutics at the Institute of Cancer Research (ICR). HN5 and MCF7 cells were grown in Dulbecco's Modified Eagle Medium (DMEM) supplemented with 10% Foetal Bovine Serum (FBS) (GIBCO) and 50 units mL^{-1} of penicillin-streptomycin (GIBCO) and cultured in a 37 °C humidified incubator with 5% CO_2. MDA-MB-468 and SKBR3 cells were provided by Dr Larissa Lezina at the Department of Genetics and Genome Biology, University of Leicester. MDA-MB-468 and SKBR3 cells were grown in Roswell Park Memorial Institute (RPMI) 1640 medium supplemented with 10% Foetal Bovine Serum (FBS) (GIBCO) and 50 units mL^{-1} of penicillin-streptomycin (GIBCO) and cultured in a 37 °C humidified incubator with 5% CO_2.

Synthesis of nanoMIPs – preparation of solid phase

The six EGFR peptides were previously identified via snapshot imprinting.[1] Peptide-0 was introduced as a scrambled version of peptide-1 in order to assess specificity of binding and cell response. A terminal cysteine group was added to each peptide to facilitate immobilisation on a solid phase with, a glycine group as a spacer (listed in Table 1 in parentheses).

Solid phase synthesis of molecularly imprinted polymers was carried out as previously described [2]. Glass beads (60 g) were boiled in NaOH (1 M, 100 mL) for 15 min, washed with water (5 × 200 mL), PBS (100 mL) (PBS), water (5 × 200 mL) and acetone (2 × 100 mL). They were then dried by heating to 120° C for 30 min. A solution of 2% (v/v) (3-iodopropyl)trimethoxysilane (IPTMS) in dry toluene

(60 mL) was added to the activated beads. Following overnight (16 h) incubation at 70 °C, the beads were washed with acetone (4 × 100 mL) and dried at 120 °C for 30 min. EGFR peptide (5 mg) was dissolved in borate buffer (pH 9.2, 30 mM sodium tetraborate, 25 mL), added to the IPTMS-functionalised glass beads (60 g) and incubated overnight. Mercaptoethanol (20 µL, 0.3 mmol) was then added and incubated for 2 h. The beads were then washed with water (3 × 200 mL) and acetone (1 × 100 mL) and allowed to dry.

Synthesis of nanoMIPs

Two batches of nanoMIPs were made for each peptide: fluorescent nanoMIPs for imaging analysis and non-fluorescent nanoMIPs for biochemical investigations.

A monomeric mixture was prepared consisting of N-isopropylacrylamide (NIPAm) (19.5 mg, 0.18 mmol), N-tert-butylacrylamide (TBAm) (15 mg, 0.126 mmol) in 200 µL of ethanol, N,N'-methylene-bis-acrylamide (MBAA) (3 mg, 19.5 µmol), acrylic acid (1.1 µL, 16.0 µmol), N-(3-aminopropyl)methacrylamide hydrochloride (3 mg, 16.8 µmol) dissolved in 50 mL of water. For the preparation of fluorescent nanoMIPs, the monomeric mixture included the addition of fluorescein acrylamide monomer (1 mg) dissolved in 0.2 mL of ethanol. Peptide-functionalised glass beads (60 g) were then added, and polymerisation was initiated by via the addition of potassium persulfate (KPS) (12 mg, 44.4 µmol) and N,N,N',N'-tetramethylethylenediamine (TEMED) (6 µL, 40.3 µmol) dissolved in PBS (400 µL). The polymerisation was carried out for 1 h at 20 °C.

The solid phase was first washed with room temperature water (4 × 25 mL) in order to remove low-affinity polymers and non-polymerised monomers. Elution of high affinity nanoMIPs was carried out by washing the solid phase with 45 °C ethanol (4 × 25 mL). The collected ethanol fraction was evaporated using a rotor evaporator and reconstituted with HPLC-grade water following the dialysis against water in the Snakeskin dialysis tubes (22 mm, 10 kDa pore size) (Life Technologies Ltd. (Invitrogen), UK). The dialysed nanoMIPs were lyophilised and stored at 4 °C until further use.

Dynamic light scattering (DLS)

DLS analysis was performed using a Zetasizer Nano (Nano-S) from Malvern Instruments Ltd (Malvern, UK), equipped with a 633 nm laser. MIPs were diluted to 10 µg mL^{-1} in water. Five measurements, each consisting of 13 runs, were performed on each sample at room temperature.

Surface plasmon resonance (SPR) for characterisation of EGFR - nanoMIPs interactions

Binding analysis was performed using a Biacore 3000 instrument (Cytiva, UK) at 25 °C using PBS as the running buffer at a flow of 35 µL min^{-1}. The self-assembled gold sensor chip was plasma-cleaned using a K1050X RF Plasma Etcher/Asher/Cleaner barrel reactor (Quorum Technologies Ltd, Lewes,

UK) and placed in a solution of mercaptododecanoic acid in ethanol (1.1 mg mL^{-1}) where they were stored until use. Before assembly, the sensor chip was rinsed with ethanol and water and dried in a stream of air. Peptide-0 and peptide-1 were immobilised in situ on the chip surface containing carboxyl groups using thiol coupling. First, the surface was activated using an EDC and NHS mixture (0.4 mg and 0.6 mg mL^{-1}, respectively). 2-(2-pyridinyldithio)ethaneamine hydrochloride (PDEA, 80 mM) in 50 mM sodium borate buffer (pH 8.5) was injected in order to introduce disulphide bonds; 100 μL of 10 μg mL^{-1} peptide solution in PBS was then injected at a 15 μL mL^{-1} flow rate followed by surface deactivation using a cysteine/NaCl solution. The peptide-specific MIPs were briefly sonicated and diluted with PBS in the concentration range of 0.04 - 1 nM. Sensorgrams were collected sequentially for all analyte concentrations running in KINJECT mode (injection volume - 100 μL and dissociation time - 120 sec). Dissociation constants (Kd) were calculated from plots of the equilibrium biosensor response using the BiaEvaluation v4.1 software using a 1:1 Langmuir binding model fitting after subtraction of drift and bulk components.

Fluorescence microscopy

Cells were seeded in whole media in 8-well Nunc Lab-Tek Chamber Slides to a concentration of 50,000 cells per 0.7 cm^2 well. The slides were incubated at 37 °C and 5% CO_2 overnight, at which point they approached confluency. The cells were then washed with 37 °C PBS and treated with solutions of MIPs (0.1 mg mL^{-1}) in whole media. The slides were then incubated at 37 °C and 5% CO_2 for 2 h, after which they were washed three times with room temperature PBS, fixed with 4% PFA in PBS for 15 min at room temperature, washed once more with room temperature PBS, and mounted using pre-made DAPI/antifade mounting solution (Sigma-Aldrich). The slides were then imaged using an Olympus BX51 fluorescence microscope (Olympus Life Sciences, Tokyo, Japan), using the FITC optical filter setting.

Confocal microscopy

MDA-MB-468 cells were plated on round cover slips in a 24-well plate (approximately 50–60% confluence) in full growth medium. The next day, the medium was changed to serum-free and incubated for 24 h. Next, the cells were incubated in a serum-free medium supplemented with MIP-0, MIP-1, MIP-2 or MIP-5 at a concentration of 100 μg mL^{-1} for 1 h before being washed with PBS. Cells were then incubated with EGF in PBS (2 ng mL^{-1}) for 15 min. The cells were then rinsed twice with PBS, chemically fixed by treatment with 4% (w/w) PFA in PBS for 15 min at room temperature, and rinsed again three times with PBS. The cells were then permeabilised and blocked via incubation in 0.1% Triton X-100 and 2% (w/w) BSA in PBS for 30 min. Cells were washed again three times with PBS, incubating for 5 min each time. Staining with primary antibody was performed with anti-human EGFR antibody at a dilution of 200× in PBS, incubated overnight at 4 °C. The cells were then washed three

times with PBS, and stained with secondary antibody at a dilution of 500× in 2% BSA in PBS for 60 min at room temperature, protected from light. The cells were then washed three times with PBS, and counter staining with DAPI in PBS (300 nM) for 10 min at room temperature. Cells were washed three times with PBS, and mounted onto glass microscope slides using mounting medium.

Flow cytometry

Cells were seeded to approximately 3 million cells per T25 flask. NanoMIPs were added to a concentration of 100 μg mL^{-1}, and the cells incubated at 37 °C and 5% CO_2 either for 3 h or overnight. The cells were then washed three times with PBS, detached using trypsin and suspended in ice-cold BSA in PBS (0.5% w/w) with approximately 10^6 cells per sample tube. The samples were then analysed using a FACS Canto A Flow Cytometer (BD Biosciences, Berkshire, UK) using the FITC optical filter setting.

Assessment of EGFR phosphorylation

MDA-MB-468 cells were seeded on a 6 cm Petri dish to a confluence of 60% in full growth medium. The following day, the medium was changed to a serum-free equivalent and the cells were incubated for an additional 24 h to exhaust the intracellular supply of growth factors. Each of the EGFR-MIPs were sonicated in an ultrasonic bath at 25 °C for 10 min then added to cells at a concentration of 100 μg mL^{-1} in serum-free medium. MDA-MB-468 cells without MIPs were used as a control. Cells incubated with EGF at a concentration of 10 ng mL^{-1} were used as a positive control for EGFR activation. After incubation, the cells were washed with PBS once and lysed for 20 min on ice using RPIA buffer supplemented with protease inhibitors (Roche, Hertfordshire, UK). Subsequently, the cell lysates were briefly sonicated and subjected to SDS-PAGE. The following antibodies were used in WB: anti-EGFR, pEGFR (Y1068), pAKT(S473), pERK(T202) (Cell Signaling Technology, US) and secondary antibodies conjugated with horseradish peroxidase (Sigma Aldrich). The signal was visualised with enhanced luminol-based chemiluminescent substrate (Thermo Fisher Scientific Ltd, Loughborough, UK) using the Chemidoc Touch Imaging System (BioRad, Watford, UK).

shRNA-mediated knockdown of EGFR in H1299 cells

Lentiviral pLKO.1 plasmid expressing EGFR shRNA was obtained from Open Biosystems (TRCN0000121069) via Thermo Fisher Scientific (Loughborough, UK). H1299 cells were infected overnight with lentivirus expressing shRNA-EGFR. Successful clones were selected for resistance to puromycin using the growth medium supplemented with 1.6 μg/ml puromycin for subsequent propagation.

S2. Cellular uptake study

The previously described nanoMIPs were assessed with regards to cellular uptake. Two cell lines exhibiting high expression levels of EGFR were selected: HN5 (squamous cell carcinoma of the tongue) and MDA-MB-468 (adenocarcinoma of the breast). These cell lines were previously mapped using snapshot imprinting in order to identify epitopes of EGFR suitable for targeting with nanoMIPs [1]. These cells were treated with fluorescent batches of MIP-1 to MIP-6 as described above. Additionally, a nanoMIP was prepared against a scrambled variant of peptide-1 in order to assess EGFR specificity (MIP-0 and peptide-0, respectively).

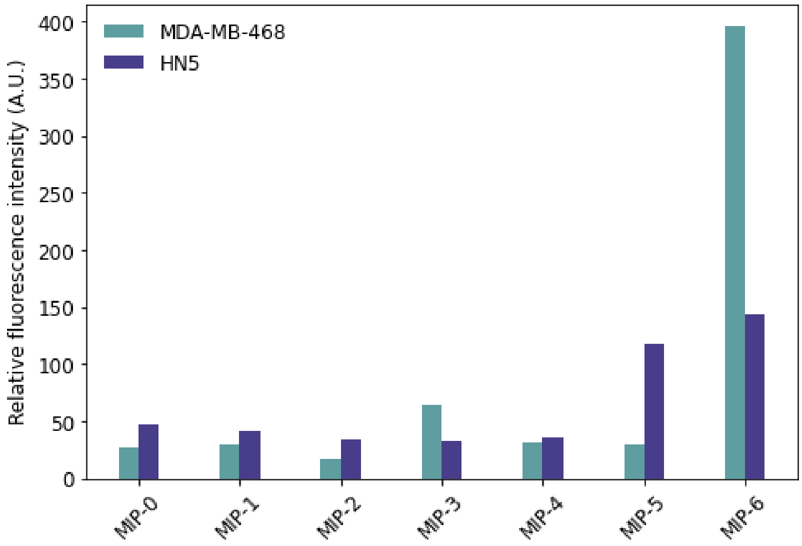

Figure S1. Relative fluorescence intensity of MDA-MB-468 and HN5 cells treated with MIP-0 to MIP-6.

As shown in Figure S1, only MIP-5 (in the case of HN5) and MIP-6 (for both HN5 and MDA-MB-468) showed significantly elevated fluorescence levels in comparison to the control MIP-0. This implies that in the majority of cases, nanoMIP binding was primarily via non-specific interactions. Notably, the MIP with the highest level of binding, MIP-6, was imprinted with an epitope from the EGF binding domain of EGFR. Additionally, MIP-6 shows higher binding to HN5 than MDA-MB-468, and the inverse is observed for MIP-5. Given the complex environment of the cell surface, different epitopes of surface proteins such as EGFR can be differentially exposed across multiple cell lines and in the presence of oncogenic mutations.[2,3] This can allow for more specific approaches to targeted imaging and drug delivery by rational selection of epitopes. MIP-6 was used for fluorescent imaging of both cell lines, and demonstrated the formation of clusters of nanoparticles (Figure S2).

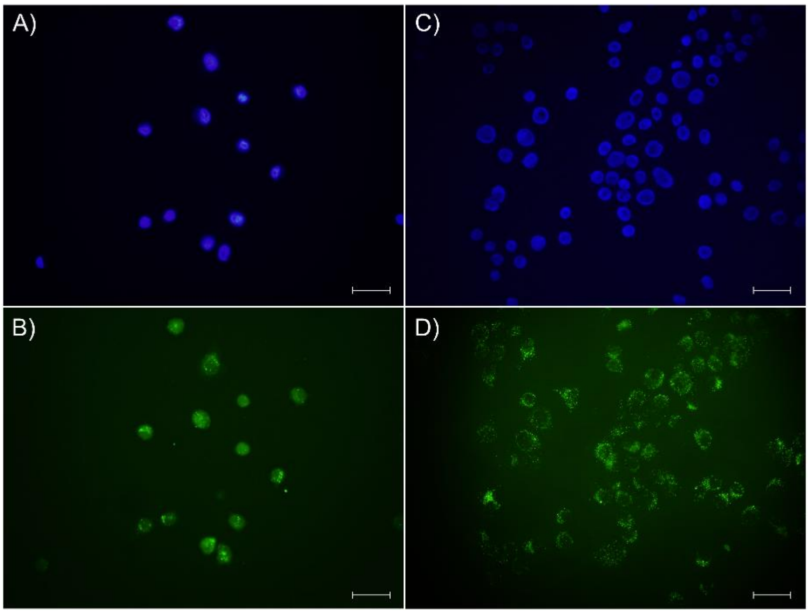

Figure S2. Fluorescence microscopy of cells treated with fluorescent MIP-6. A) MDA-MB-468 DAPI stain; B) MDA-MB-468 MIP-6; C) HN5 DAPI stain; D) HN5 MIP-6. Scale bar = 20 µm.

Since MIP-6 showed stronger binding to EGFR compared to MIP-5 we decided to investigate the downstream effects of MIP-6. Thus, we carried out MTT test on two breast cancer cell lines differing in the EGFR status: MDA-468 (high EGFR) and SKBR-3 (low EGFR), non-treated or treated with MIP-6 (**Figure S3**). The high dose treatment (100 µg) with MIP-6 attenuated the proliferation rate of MDA-468 cells (high EGFR) down to 50% of the control value leaving the level of proliferation of SKBR-3 cells (low EGFR) almost intact (85% of the control level). This result emphasizes the importance of extracellular nanoMIPs for EGFR-mediated signal transduction.

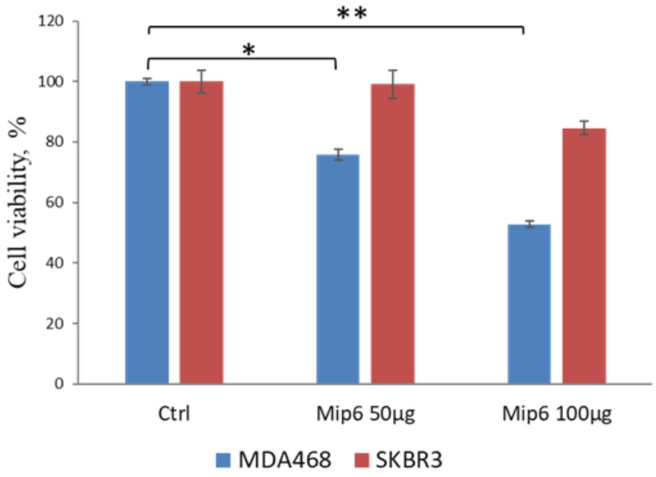

Figure S3. Effect of nanoMIP 6 on cell viability of MDA-MB-468 and MCF7 cell lines. Mean ± SEM. Statistical significance was calculated by one-way ANOVA compared to the control sample, *P < 0.05, ** P < 0.005.

That MIP-0 could induce autophosphorylation of EGFR through nonspecific interactions prompted us to address the question of whether such nanoMIPs can promote the growth of tumor cells?

To address this question, we incubated three cancer cell lines: MDA-468 (breast cancer, high level of EGFR), H1299 wt (lung cancer, low level of EGFR) and H1299 derivative cells with shRNA-mediated knockdown of EGFR (KD EGFR) with MIP-0 and MIP-2 with either lack of or high affinity to EGFR, respectively (**Figure S4**). The results obtained clearly indicated that incubation of cells with non-specific MIP-0 only marginally increased proliferation of MDA-468 and H1299 cells. In contrast, MIP-2 significantly attenuated the proliferation level of MDA-468 cells without affecting H1299 cells irrespective of their EGFR status (Figure S4). Apparently, the level of EGFR expression in H1299 is too low to be detected by nanoMIPs. These results also indicate that the effect on autophosphorylation of EGFR does not necessarily translate into the alteration of growth and that other molecular events (e.g. intracellular localization) may be critical for the *in vivo* effects.

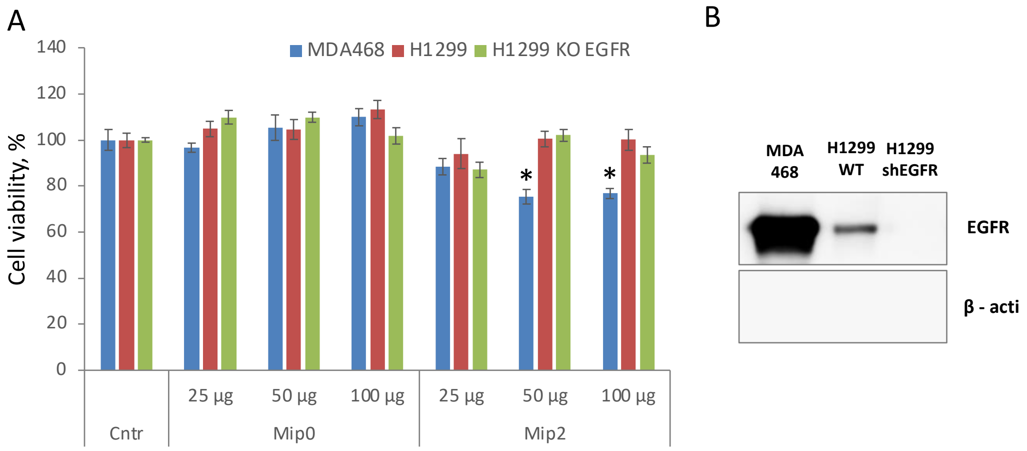

Figure S4. Effect of different MIPs on viability of cell lines depending on the level of total EGFR+. A) MTT-test, demonstrating cell viability of MDA468, H1299 (containing significantly less EGFR, then in MDA468 cell line) and H1299 KO EGFR (EGFR absence) after 48 hours treatment with Mip0 and Mip2. Mean ± SEM. Statistical significance was calculated by one-way ANOVA compared to the control sample, *P < 0.05. B) A comparative analysis of total levels of EGFR in MDA468, H1299, and H1299 KO EGFR cell lines by Western blotting with anti-EGFR serum.

Table S1. Physical characterisation of nanoMIPs.

NanoMIP	Diameter, nm	K_D, nM (χ2)
MIP-1	144 ± 10	47.1 (3.36E-05)
MIP-2	154 ± 8	0.2 (208)
MIP-3	184 ± 10	22.2 (3.03E-05)
MIP-4	145 ± 9	31.9 (4.15E-05)
MIP-5	149 ± 11	11 (0.02)
MIP-6	143 ± 11	16.8 (0.4)

References

(1) Piletsky, S. S.; Piletska, E.; Poblocka, M.; Macip, S.; Jones, D. J. L.; Braga, M.; Cao, T. H.; Singh, R.; Spivey, A. C.; Aboagye, E. O.; Piletsky, S. A. Snapshot Imprinting: Rapid Identification of Cancer Cell Surface Proteins and Epitopes Using Molecularly Imprinted Polymers. *Nano Today* **2021**, *41*, 101304. https://doi.org/10.1016/J.NANTOD.2021.101304.

(2) Gan, H. K.; Burgess, A. W.; Clayton, A. H. A.; Scott, A. M. Targeting of a Conformationally Exposed, Tumor-Specific Epitope of EGFR as a Strategy for Cancer Therapy. *Cancer Res* **2012**, *72* (12), 2924–2930. https://doi.org/10.1158/0008-5472.CAN-11-3898.

(3) Garrett, T. P. J.; Burgess, A. W.; Gan, H. K.; Luwor, R. B.; Cartwright, G.; Walker, F.; Orchard, S. G.; Clayton, A. H. A.; Nice, E. C.; Rothacker, J.; Catimel, B.; Cavenee, W. K.; Old, L. J.; Stockert, E.; Ritter, G.; Adams, T. E.; Hoyne, P. A.; Wittrup, D.; Chao, G.; Cochran, J. R.; Luo, C.; Lou, M.; Huyton, T.; Xu, Y.; Fairlie, W. D.; Yao, S.; Scott, A. M.; Johns, T. G. Antibodies Specifically Targeting a Locally Misfolded Region of Tumor Associated EGFR. *Proc Natl Acad Sci U S A* **2009**, *106* (13), 5082–5087. https://doi.org/10.1073/PNAS.0811559106.
